# Randomized Clinical Trial: Probiotics Alleviated Oral-Gut Microbiota Dysbiosis and Thyroid Hormone Withdrawal-Related Complications in Thyroid Cancer Patients Before Radioiodine Therapy Following Thyroidectomy

**DOI:** 10.3389/fendo.2022.834674

**Published:** 2022-03-08

**Authors:** Baiqiang Lin, Fuya Zhao, Yang Liu, Xin Wu, Jing Feng, Xiangren Jin, Wei Yan, Xiao Guo, Shang Shi, Zhiyong Li, Lujia Liu, Hongye Chen, Haoran Wang, Shuang Wang, Yu Lu, Yunwei Wei

**Affiliations:** ^1^ Department of Oncology and Laparoscopy Surgery, The First Affiliated Hospital of Harbin Medical University, Harbin, China; ^2^ Department of Pancreatic and Gastrointestinal Surgery Division, HwaMei Hospital, University of Chinese Academy of Science, Ningbo, China; ^3^ Department of Nuclear Medicine, The First Affiliated Hospital of Harbin Medical University, Harbin, China

**Keywords:** radioiodine therapy, complications, oral microbiota, gut microbiota, probiotics, thyroid hormone withdrawal

## Abstract

**Background:**

Thyroid hormone withdrawal (THW) in postoperative thyroid cancer patients who need always accompanied by complications (e.g., dyslipidemia and constipation). At present, there are no effective and safe means to alleviate these complications.

**Purpose:**

We aimed to assess the oral-gut microbiota profiles in THW patients then investigate whether probiotics could alleviating alleviate THW related complications and investigate whether these therapeutic effects were associated with the oral-gut microbiota state.

**Methods:**

Fifty eligible thyroid carcinoma patients undergoing thyroidectomy were randomly assigned to receive probiotics or placebo during THW. Complications were assessed through validated questionnaires and plasma lipid indicators. The complex probiotics preparation was composed of *Bifidobacterium infantis*, *Lactobacillus acidophilus*, *Enterococcus faecalis*, and *Bacillus cereus*.

**Results:**

Probiotics alleviated lack of energy, constipation, weight gain, and dry mouth and decreased the levels of fecal/serum LPS and plasma lipid indicators (total cholesterol, triglycerides, low-density lipoprotein, and apolipoprotein A) (P < 0.05). Gut and oral microbial diversity were significantly decreased after THW, while an increased microbial dysbiosis index (MDI) was observed. Probiotics distinctly restored the gut and oral microbial diversity. Increased *Holdemanella*, *Enterococcus*, and *Coprococcus_2*, while decreased *Fusobacterium*, *Eubacterium_ruminantium_group*, *Ruminococcus_1*, and *Parasutterella* in the gut were found after probiotics intervention. Lack of energy, constipation, weight gain, and dyslipidemia were seen to be related to the above microbiota. In addition, probiotics reduced oral *Prevotella_9*, *Haemophilus*, *Fusobacterium*, and *Lautropia*, which were positively correlated with the occurrence of dry mouth.

**Conclusion:**

Probiotics reduce the incidence of complications in patients after THW, which may be related to modifying the oral and gut microbiota.

**Clinical Trial Registration:**

[https://clinicaltrials.gov/], identifier America Clinical Trial Registry NCT03574051.

## Introduction

Thyroid cancer (TC) is the most prevalent malignant neoplasm in the endocrine system. The incidence of TC has been increasing in the past 25 years (1). The majority of TC patients are differentiated thyroid cancer (DTC) developing from thyroid follicular cells and are classified as papillary or follicular carcinomas. Radioactive iodine (RAI) has been administered following thyroidectomy in patients with DTC for remnant ablation and adjuvant treatment (2). In patients receiving RAI treatment, the uptake of iodine depends on the stimulation of thyrotropin (TSH), which can be achieved by thyroid hormone withdrawal (THW) or injecting recombinant human thyroid-stimulating hormone (rhTSH) (3). Compared with rhTSH injection, THW therapy is low-cost and widely used in Asian countries (4, 5). However, THW always is accompanied by various complications, including fatigue, constipation, weight gain, edema, and hypercholesterolemia (6–9), which have an obvious negative impact on the patients’ quality of life (9, 10). Thus, investigating the mechanism for THW related symptom is help to benefit the thyroid cancer patients who need to receive RAI after surgery. Although DTC patients who received recombinant human thyrotropin (rhTSH) to maintain normal thyroid hormone (free triiodothyronine (fT3), free thyroxine (fT4)) levels, these complications still occur (11, 12). Thus, the THW related complications cannot be all be blamed for hypothyroidism, while some other factors should be taken into consideration. Previous studies have shown that constipation, weight gain, fatigue, and dry mouth attribute to the oral and gut microbiota perturbation (13–15). There is accumulating evidence indicating a thyroid-gut axis exists (16, 17). It appears to display an interaction effect between the gut microbiota and thyroid function (16, 18). Therefore, we suspect that THW may cause oral and gut microbiota dysbiosis then induce these complications.

Since our previous study showed the gut microbiota of DTC patients was already disordered (19). This study aims to figure out the gut and oral microbiota characters in postoperative DTC patients after THW. Then, we perform an intervention study to investigate the beneficial effects of probiotics on alleviating the complications of DTC patients with THW.

## Materials and Methods

### Ethics

The authors ensure that the current clinical trial has been carried out by the Code of Ethics of the World Medical Association (Declaration of Helsinki). This study was a randomized, parallel‐group, double‐blind, placebo-controlled, adaptive randomized controlled trial (RCT). The intervention period was four weeks, while the primary outcome was assessed at week 4. The Ethics Committee approved all protocols applied in this study at the First Affiliated Hospital of Harbin Medical ·University (Eth. 201816). All patients gave written informed consent for participation in the study. In addition, a clinical study was registered with the America Clinical Trial Registry (NCT03574051).

### Study Design and Patient Enrollment

**Figure 1:**
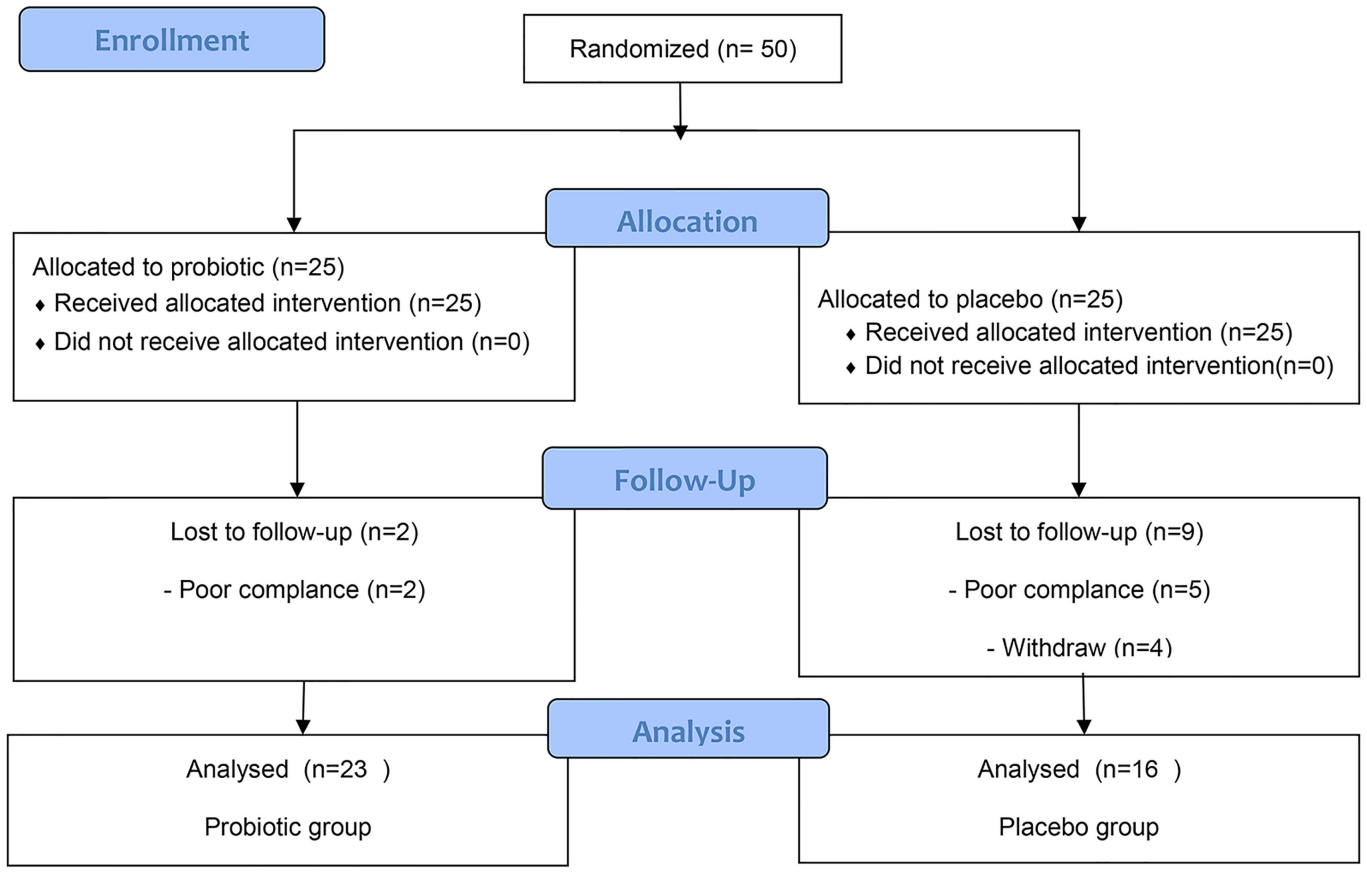
The CONSORT diagram.

Based on the hypothesis that the average incidence of complications of lack of energy in the placebo group and the probiotic group would be 63.4% and 21% (10), respectively, 32 patients were needed (2 - sided α = 0.05, 1 - β = 0.9, and 1:1 ratio). A sample size of 32 was calculated, considering drop-out expected for the follow−up study; the total sample size calculated was 50. We recruited a total of fifty post-thyroidectomy DTC patients awaiting THW therapy from the Department of Nuclear Medicine of the First Affiliated Hospital of Harbin Medical University between January 2017 and April 2018 (**Figure 1**). The inclusion criteria for patients were as follows: 18 to 65 years of age; differentiated thyroid cancer patients who had undergone total thyroidectomy before radioiodine therapy and awaited THW treatment; four weeks of levothyroxine withdrawal after surgery to achieve TSH elevation above 30 mIU/mL; consented to the 4-week probiotic treatment; agreed for serum lipid testing, because this was not routinely performed in our institute. The patient was told to take a low-iodine diet during THW. The random allocation of patients is carried out by a random number code generated by the computer of Harbin Medical University. The patients were randomly divided into 6 blocks with a ratio of 1:1 and received probiotics or a placebo (only the size of the block is known to the statistician). The medicines are distributed and packaged according to random numbers. The parameter description files such as the random number seed blind code, block length, and random number are sealed in the First Affiliated Hospital of Harbin Medical University. Throughout the trial period, the blinding code was not disclosed. The probiotics (Bifidobacterium Tetravaccine Tablets, Hangzhou Yuanda Biopharmaceutical Co., Ltd., SFDA approval number: S20060010; containing > 10^6^ CFU/tablet *B. infantis*, > 10^6^ CFU/tablet *L. acidophilus*, > 10^6^ CFU/tablet *E. faecalis*, > 10^5^ CFU/tablet *B. cereus* and > 10^6^ CFU/tablet total bacteria). Probiotic or placebo (starch) was supplied from the beginning to the End of treatment for up to 4 weeks (3 capsules two times a day). The shape, color, and other characteristics of the placebo are designed according to the Chinese Pharmacopoeia standards and are the same as the shape and color of the probiotic composition. Probiotics and placebo capsules were randomly numbered, and blinding codes were not disclosed to patients or treating physicians. Finally, 23 patients were included in the probiotics group, and 16 patients were included in the placebo group (**Figure 1**).

### Clinical Outcomes

The primary outcome was a change in the incidence of complications after four weeks between probiotics and placebo. The secondary clinical outcomes included oral and gut microbiota, plasma lipid levels, thyroid function, and liver function. The severity of complications was assessed by the Thyroid symptom questionnaire (TSQ) (10), focusing on the following items: lack of energy/fatigue (TSQ-LOE), constipation (TSQ-C), edema (TSQ-E), weight gain (TSQ-WG), and dry mouth (TSQ-DRY) (6, 7). The TSQ was developed based on a previous study (10) and included questions on Scores on a Likert scale were defined as 0-absent, 1-complications absent, 2-rarely present, 3-present, and 4-severely present. A single research assistant administered the questionnaire to all the volunteers.

### Sample Collection and Clinical Parameters

For the enrolled patients, we conducted longitudinal sampling, Baseline: 2-4 days after total thyroidectomy (normal thyroid function), End of intervention: 4 weeks after THW treatment (Levothyroxine withdrawn)/THW plus probiotics. Plasma, fecal, and oral washings samples were obtained from each subject (B-THW (before the treatment of THW plus a placebo) group (n = 16), B-THW-P (before the treatment of THW plus the probiotic combination) group (n = 23), A-THW (after treatment with THW plus a placebo) group (n = 16), and A-THW-P [after treatment with THW plus the probiotic) group (n = 23)]. The specific collection and processing methods and a comprehensive description of the clinical parameter analysis are provided in the **Supplementary Material**. Plasma samples were collected for thyroid function detection (free triiodothyronine[fT3], free thyroxine[fT4], and thyroid-stimulating hormone [TSH]) and thyroglobulin [TG], liver function (alanine aminotransferase [ALT], aspartate transaminase [AST], album [ALB], total protein [TP], globulin [GLB), total bilirubin [TBIL], direct bilirubin [DBIL], indirect bilirubin [IBIL], gamma-glutamyl transpeptidase [GGT], alkaline phosphatase [AKP], lactate dehydrogenase [LDH], blood urea nitrogen [BUN], creatinine [Cr], uric acid [UA], glucose [GLU]), plasma lipid (total cholesterol [CHOL], triacylglycerol [TG], low-density lipoprotein [LDL], apolipoprotein A [Apo A], high-density lipoprotein [HDL], very low density lipoprotein [VLDL], apolipoprotein B [Apo B], lipoprotein(a) [Lpa]) and plasma LPS analyses; fecal and oral washings samples were used for 16S rRNA gene sequence and fecal LPS detection.

### gDNA Extraction and 16S rRNA Gene Sequencing

DNA isolation on all fecal samples was performed the same, using the AllPrep DNA/RNA Mini kit (TIANGEN Biotech, Beijing, China). DNA isolation on oral washings was performed with the Ultraclean Microbial DNA isolation kit (MO BIO, Carlsbad, California, USA). The V3-V4 region of the bacterial 16S rRNA gene was amplified and sequenced using an Illumina HiSeq2500 platform (Illumina, California, USA). Raw sequence data were uploaded to the National Center for Biotechnology Information (NCBI) database and are available through accession number PRJNA784752.

### Statistical Analyses

The clinical parameters and linear correlation analysis were performed using the Statistical Package for the Social Sciences (SPSS) version 22.0. Alpha and beta diversity analyses were calculated in our samples using Quantitative Insights Into Microbial Ecology (QIIME, Version 1.7.0) based on rarefied operational taxonomic unit (OTU) count and displayed using R software (Version 2.15.3).

A differential prevalence analysis was performed using the Wilcoxon rank-sum test at the genus levels. The analyses were restricted to taxa with a prevalence > 50% and a minimal proportion > 0.002, and only taxa with P < 0.05 were considered statistically significant. The predicted functional composition profiles were collapsed into KEGG pathways (level 3) based on 16S rRNA sequences using PICRUSt. Correlations among the variables (clinical parameters, different microbiota, etc.) were computed using Spearman rank correlation, and the correlation (Student’s t-test, P < 0.05, |correlation coefficient| > 0.3) are presented using a heatmap or network diagram (Cytoscape, Version 3.2.1) (20).

## Results

### Baseline Characteristics Examined for All Volunteers

Between January 2018 and April 2019, 50 patients were randomly assigned to either the probiotics or placebo groups. Seven patients who received at least one dose of the drug were automatically withdrawn from the study (the probiotics/placebo group, n = 2/n = 5) because of a failure to undergo follow-up or at their request. In addition, another four patients were excluded from the protocol set because of poor drug compliance in the placebo group (**Figure 1**). All patients were thoroughly informed about their diseases and the treatments they would receive. Their sex, age, baseline characteristics, and tumor classification are summarized in **Table 1**. Twenty-three patients were designated as the probiotics group (n = 23), and 16 patients were assigned as the placebo group (n = 16) (**Figure 1**). The treatment groups were well balanced, and there was no marked difference between the two groups.

**Table 1:** Clinical and demographic features.

Characteristics	Placebo group (n=16)	Probiotic group (n=23)
Sex (M/F)	4/12	2/21
Age (years, mean ± SD)	41.38 ± 7.07	39.13 ± 9.15
BMI (kg/m^2^, mean ± SD)	24.11 ± 3.48	23.70 ± 2.93
Tumor stage, No. (%)		
T1	4	7
T2	6	8
T3	6	8
T4	0	0
Node stage, No. (%)		
N0	0	0
N1	12	14
N2	4	9
N3	0	0

BMI, Body Mass Index; SD, standard deviation.

### The Probiotic Reduced the Incidence of Complications and Plasma Lipid Levels

As shown in **Table 2**, our data also indicated that the probiotics reduced the incidence of complications (the percentage of subjects with complications [score 3 and 4 clubbed together]), TSQ-C (62.5% vs. 8.7%, P = 0.004), TSQ-LOE (62.5% vs. 30.4%, P = 0.047), TSQ-WG (68.8% vs. 34.8%, P = 0.037), and TSQ-DRY (68.8% vs. 30.4%, P = 0.018) in A-THW-P group, compared with those of the A-THW group. However, the incidence of TSQ-E was not significantly different between the two groups (P > 0.05, **Table 2**). The effect size of reduction in complications scores between two groups is shown separately in **Table 2**. To assess whether the probiotics alleviated dyslipidemia, we monitored the plasma lipid indexes in the patients. We found that the probiotics significantly reduced the total cholesterol (CHOL) index (5.57 ± 0.99 vs. 6.56 ± 1.15, P = 0.006), triglyceride (TG) index (1.79 ± 0.68 vs. 2.41 ± 0.66, P = 0.014), low-density lipoprotein (LDL) index (3.80 ± 0.70 vs. 4.36 ± 0.55, P = 0.017), and apolipoprotein A (Apo A) index (1.56 ± 0.18 vs. 1.68 ± 0.05, P = 0.001) values compared with those of the A-THW group (**Table 3**). Moreover, the probiotics restored the rates of the CHOL index (65% vs. 25%), TG index (74% vs. 38%), LDL index (61% vs. 31%), and Apo A index (74% vs. 31%) in the A-THW-P group to normal levels in comparison with the A-THW group (**Table 4**). Although probiotics were found to slightly increase free triiodothyronine (fT3) levels compared to the A-THW group, there was no statistical difference (1.21 ± 0.25 vs. 1.31 ± 0.29, P = 0.052). However, the probiotics did not affect thyroglobulin and liver function indicators compared with the A-THW group (P > 0.05, **Table 3** and **Supplementary Table 1**).

**Table 2:** The percentage of subjects with complications (score 3 and 4 clubbed together) and the mean thyroid symptoms score.

Variable	A-THW group (n=16)	A-THW-P group (n=23)	P1 value (The percentage of complications)	P2 value (The symptoms score)
Constipation (TSQ-C)	62.5% (2.69 ± 0.98)	8.7% (1.57 ± 0.88)	0.004	0.001
Lack of energy (TSQ-LOE)	62.5% (2.88 ± 1.05)	30.4% (1.78 ± 1.06)	0.047	0.005
Weight gain (TSQ-WG)	68.8% (2.88 ± 0.93)	34.8% (1.91 ± 1.21)	0.037	0.013
Dry mouth (TSQ-DRY)	68.8% (2.94 ± 0.90)	30.4% (1.87 ± 1.19)	0.018	0.007
Edema (TSQ-E)	26.1% (2.20 ± 1.05)	43.8% (2.00 ± 0.83)	0.250	0.641

All complications (score 3 and 4 clubbed together) are represented as a percentage. All the complications scores are in values (mean ± SD). TSQ, Thyroid symptom questionnaire, P1 Value of the percentage of complications between A-THW vs. A-THW-P group; P2 Value of the complications score between A-THW vs. A-THW-P group; SD, Standard deviation. P-value < 0.05 was considered significant. A-THW, after treatment with THW plus a placebo; A-THW-P, after treatment with THW plus the probiotic.

**Table 3:** Plasma indicators.

Variable	Placebo group (n=16)	Probiotic group (n=23)	P value
Plasma lipid			
CHOL (µmol/L, mean ± SD)			
Baseline	4.68 ± 0.49	4.68 ± 0.47	0.703
End of intervention (week 4)	5.57 ± 0.99	6.56 ± 1.15	0.006*
TG (µmol/L, mean ± SD)			
Baseline	1.20 ± 0.35	1.31 ± 0.42	0.417
End of intervention (week 4)	1.79 ± 0.68	2.41 ± 0.66	0.014*
LDL (µmol/L, mean ± SD)			
Baseline	3.18 ± 0.22	3.20 ± 0.30	0.966
End of intervention (week 4)	3.80 ± 0.70	4.36 ± 0.55	0.017*
ApoA (µmol/L, mean ± SD)			
Baseline	1.43 ± 0.06	1.46 ± 0.07	0.069
End of intervention (week 4)	1.56 ± 0.30	1.68 ± 0.19	0.001*
HDL (µmol/L, mean ± SD)			
Baseline	1.10 ± 0.07	1.16 ± 0.10	0.151
End of intervention (week 4)	1.33 ± 0.21	1.36 ± 0.20	0.516
VLDL (µmol/L, mean ± SD)			
Baseline	0.28 ± 0.33	0.25 ± 0.18	0.582
End of intervention (week 4)	0.33 ± 0.12	0.34 ± 0.13	0.147
ApoB (µmol/L, mean ± SD)			
Baseline	0.94 ± 0.06	0.93 ± 0.06	0.877
End of intervention (week 4)	1.09 ± 0.19	1.21 ± 0.27	0.085
Lpa (µmol/L, mean ± SD)			
Baseline	112.29 ± 36.01	119.03 ± 84.91	0.454
End of intervention (week 4)	125.59 ± 135.63	117.85 ± 129.44	0.903
Thyroid function			
fT3 (pg/mL, mean ± SD)			
Baseline	2.84 ± 0.20	2.71 ± 0.29	0.251
End of intervention (week 4)	1.21 ± 0.25	1.31 ± 0.29	0.052
fT4 (ng/dL, mean ± SD)			
Baseline	1.05 ± 0.07	1.07 ± 0.10	0.682
End of intervention (week 4)	0.43 ± 0.05	0.43 ± 0.03	0.166
TSH (µIU/mL, mean ± SD)			
Baseline	1.79 ± 0.74	1.89 ± 0.65	0.437
End of intervention (week 4)	64.90 ± 24.93	63.26 ± 20.41	0.783
Tg (IU/mL, mean ± SD)			
Baseline	6.36 ± 4.01	6.38 ± 3.43	0.832
End of intervention (week 4)	3.92 ± 3.11	3.72 ± 2.76	0.402

The measurement data and enumeration data were statistically analyzed with t-test (or one-way ANOVA for multi-group comparison) and χ2 test, respectively. Measurement data are expressed as the mean ± SD. CHOL, total cholesterol; TG, triacylglycerol; LDL, low-density lipoprotein; ApoA, apolipoprotein A; HDL, high-density lipoprotein; VLDL, very-low-density lipoprotein; ApoB, apolipoprotein B; Lpa, lipoprotein(a); fT3, free triiodothyronine; fT4, free thyroxine; TSH, thyroid-stimulating hormone; Tg, Thyroglobulin; and SD, standard deviation. P-value < 0.05 was considered significant. *P-value < 0.05

**Table 4:** Rates of People Recovering to Normal Ranges.

Index	Normal standard	Group	People within normal range, No	Total people, No.	People within normal range, No
CHOL	3.45-5.71, µmol/L	A-THW-P	15	23	65%
		A-THW	4	16	25%
TG	0.48-2.25, µmol/L	A-THW-P	17	23	74%
		A-THW	6	16	38%
LDL	0.36-4.11, µmol/L	A-THW-P	14	23	61%
		A-THW	5	16	31%
ApoA	1.20-1.60, µmol/L	A-THW-P	17	23	74%
		A-THW	5	16	31%

CHOL, total cholesterol; TG, triacylglycerol; LDL, low-density lipoprotein; ApoA, apolipoprotein A. A-THW, after treatment with THW plus a placebo; A-THW-P, after treatment with THW plus the probiotic.

### Transitions in the Gut and Oral Microbiome of DTC Patients Following THW

**Figure 2:**
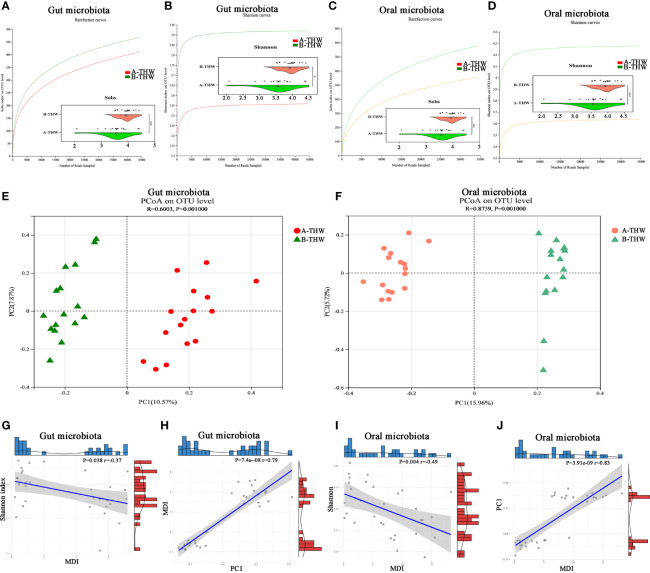
Transitions in the gut and oral microbiome of DTC patients following THW The rarefaction curve reached a plateau, indicating that the sequencing depth was adequate in B-THW (n=16) and A-THW (n=16). As estimated by the Sobs, and Shannon index, gut **(A, B)** and oral **(C, D)** microbial diversity was significantly decreased after THW treatment. The PCoA based on OTU distribution showed that the gut **(E)** and oral **(F)** taxonomic composition was significantly different after THW treatment. The relationship between the MDI and Shannon index of gut **(G)** and oral **(I)** microbiota for each sample. The relationship between the MDI and PC1 distance based on the Binary-chord of gut **(H)** and oral **(J)** microbiota for each sample. PC1, principal coordinate 1; PCoA, principal coordinate analysis; B-THW, before the treatment of THW plus a placebo; B-THW-P, before the treatment of THW plus the probiotic combination; A-THW, after treatment with THW plus a placebo; A-THW-P, after treatment with THW plus the probiotic. *P-value < 0.05; ***P-value < 0.001.

To evaluate whether THW can induce gut and oral microbiota dysbiosis, we performed 16S rRNA sequencing of fecal and oral washings samples. Rarefaction analysis showed that OTU richness in each group approached saturation (**Figures 2A–D**). As estimated by the Sobs and Shannon index, gut and oral microbial diversity were significantly decreased in the A-THW group versus the B-THW group (all P < 0.05) (**Figures 2A–D**). Beta diversity results showed a significant distinction of the gut and oral microbial communities between both groups (**Figures 2E, F**). We next calculated the microbial dysbiosis index (MDI) at the genus level (21). We found that the gut and oral microbiota of the A-THW group showed a higher MDI than that of the B-THW group (Wilcoxon rank-sum test, P < 0.001, **Supplementary Figures 1A, B**). The MDI exhibited an inverse correlation with Shannon index of alpha diversity (Spearman correlation analysis, r = -0.37, -0.49, P < 0.05, **Figures 2G, I**) and a positive correlation with PCI distance of beta diversity (Spearman correlation analysis, r = 0.79, -0.83, P < 0.05, **Figures 2H, J**). These results show a high degree of dysbiosis of DTC patients after THW in the gut and oral microbiota, consistent with the reduced bacterial diversity observed.

### Effects of Probiotics on the Gut Microbiota

**Figure 3:**
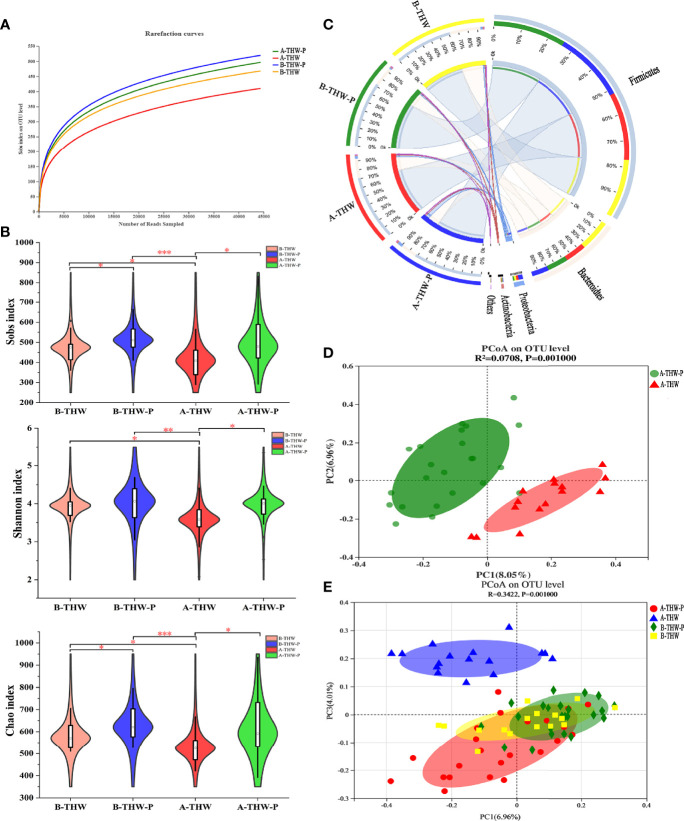
The shift in the gut microbiota architecture in patients with or without probiotic administration. **(A)** The rarefaction curve reached a plateau, indicating that the sequencing depth was adequate. **(B)** The Sobs, Shannon, and Chao indexes of the B-THW, B-THW-P, A-THW, and A-THW-P groups were compared. **(C)** In the Circos plot, the small semicircle (left half-circle) represents the species composition in the sample. The color of the outer ribbon represents the groups, the color of the inner ribbon represents the species, and the length of the ribbon represents the relative abundance of the species in the corresponding sample. The large semicircle (right half-circle) indicates the distribution proportion of species in different samples at this taxonomic level. The color of the outer ribbon represents the species, the color of the inner ribbon represents the groups, and the length of the ribbon represents the relative abundance of the species in the corresponding sample. **(D, E)** Binary-chord principal component analysis; the microbiotas of people from the B-THW, B-THW-P, A-THW, and A-THW-P groups were significantly different. B-THW, before the treatment of THW plus a placebo; B-THW-P, before the treatment of THW plus the probiotic combination; A-THW, after treatment with THW plus a placebo; A-THW-P, after treatment with THW plus the probiotic. *P-value < 0.05; **P-value < 0.01; ***P-value < 0.001.

To evaluate whether probiotics can improve microbiota dysbiosis. The microbial diversity characteristics are shown in **Supplementary Table 2**. The sequencing depths were examined by plotting the rarefaction curve of richness (Sobs index), and the curve in each group was near saturation, suggesting that the sequencing depth was adequate (**Figure 3A**). Alpha diversity was evaluated at the operational taxonomic unit (OTU) level using the Sobs, Chao, and Shannon indexes (**Figure 3B**). The results from the Wilcoxon rank-sum test revealed a significant difference in the Sobs, Chao, and Shannon indexes, which measure richness and evenness, between the B-THW and A-THW groups (**Figure 3B**, P < 0.05). With probiotic supplementation, the alpha diversity of the A-THW-P group did not show a difference compared to the B-THW-P group (**Figure 3B**, P > 0.05), which shows that the probiotic distinctly restored the gut and oral microbial diversity.

In addition, the gut microbiota structure was analyzed at the phylum level. Firmicutes, Bacteroidetes, Proteobacteria and Actinobacteria constituted the 4 most common dominant phyla in the B-THW, B-THW-P, A-THW, and A-THW-P groups (Firmicutes; 62.60%, 74.31%, 72.90%, and 74.05%, respectively; Bacteroidetes: 32.98%, 22.09%, 22.22%, and 17.54%, respectively; Proteobacteria: 2.56%, 1.71%, 2.92%, and 5.64%, respectively; Actinobacteria: 1.23%, 1.07%, 1.05%, and 1.78%, respectively, respectively); (**Figure 3C**). Moreover, the gut microbiota composition was also different among the four groups at the family and genus levels (**Supplementary Figures 2A, B**).

To assess the degree of similarity between the microbiota communities, binary-chord distance matrices were used to calculate the beta diversity values and visualize them in PCoA plots. The diversity captured by the top principal coordinates was 8.05%. The gut microbiota of patients in the A-THW-P group was separated from that of patients in the A-THW group (**Figure 3D**). Similar to the alpha diversity trend, fecal samples of patients in the A-THW-P group were closer to samples of the B-THW/B-THW-P groups than to samples of patients in the A-THW group (**Figure 3E**).

**Figure 4:**
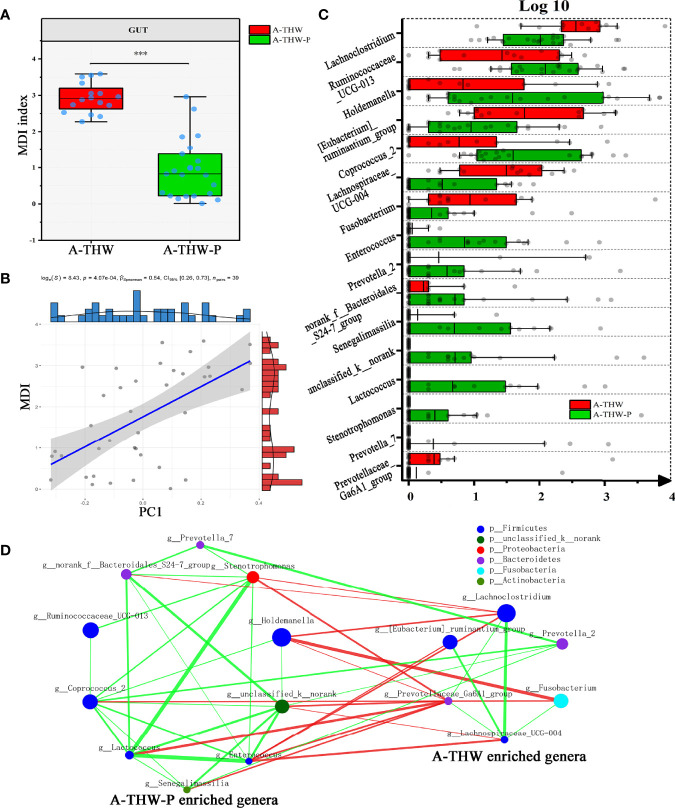
Gut microbiota phylotype alterations at the genus level in patients with or without probiotics. **(A)** Box plot showing the MDI in the A-THW, and A-THW-P groups. Significance was determined by the Kruskal-Wallis method. **(B)** Negative Pearson’s correlation between MDI and PC1 distance. **(C)** Comparisons of the relative abundance at the genus level in the A-THW, and A-THW-P groups by Mann–Whitney U-tests (P < 0.05). **(D)** A co-occurrence network was deduced from the relative abundance of 16 significantly differentially abundant genera between the A-THW and A-THW-P groups. Species are rearranged on two sides based on their enrichment in the A-THW and A-THW-P group microbiota. Spearman correlation coefficient values below −0.3 (negative correlation) are indicated as red edges, and coefficient values above 0.3 (positive correlation) are indicated as green edges. The node size shows the gene number for each species, and the node color shows their phylum-level classification. A-THW, after treatment with THW plus a placebo; A-THW-P, after treatment with THW plus the probiotic. ***P-value < 0.001.

We next combined the relevant taxa that characterized each group of patients and calculated the microbial dysbiosis index (MDI) at the genus level. We found that the gut microbiota of patients from the A-THW-P group showed a lower MDI value than that from the A-THW group (P < 0.001, **Figure 4A**). The MDI exhibited a positive correlation with the PC1 distance of beta diversity (Spearman correlation analysis, r = 0.54, P < 0.001, **Figure 4B**). These results show a lower degree of dysbiosis in the gut microbiota of patients in the A-THW-P group in contrast to the A-THW group, which is consistent with the bacterial diversity observed. The probiotics partially rescued the gut microbiota dysbiosis.

To determine the specific communities associated with patients in the A-THW-P group, we applied the Mann–Whitney U test to compare the gut microbiota at different taxon levels by confining analyses, identifying 16 differentially abundant taxa at the genus level (**Figure 4C**, P < 0.05). *Holdemanella*, *Coprococcus_2*, *Ruminococcaceae_UCG-013*, *Enterococcus*, *norank_f:Bacteroidales_S24-7_group*, *unclassified_k:norank*, *Stenotrophomonas*, *Prevotella_7*, *Lactococcus*, and *Senegalimassilia* were enriched in the patients from the A-THW-P group compared with those in the A-THW group (**Figure 4C**). In contrast, the abundances of *Lachnoclostridium, [Eubacterium]_ruminantium_group, Fusobacterium, Prevotella_2, Prevotellaceae_Ga6A1_group*, and *Lachnospiraceae_UCG-004* were decreased in patients from the A-THW-P group compared with those from the A-THW group (**Figure 4C**). Then, these 16 differentially abundance genera were applied to build an interaction network (Spearman’s correlation value < -0.3 or > 0.3, P < 0.05, **Figure 4D**). The A-THW-P group-enriched species were more highly interconnected than the A-THW group-enriched genera.

### Associations Between Gut Microbial Species and Clinical Features

**Figure 5:**
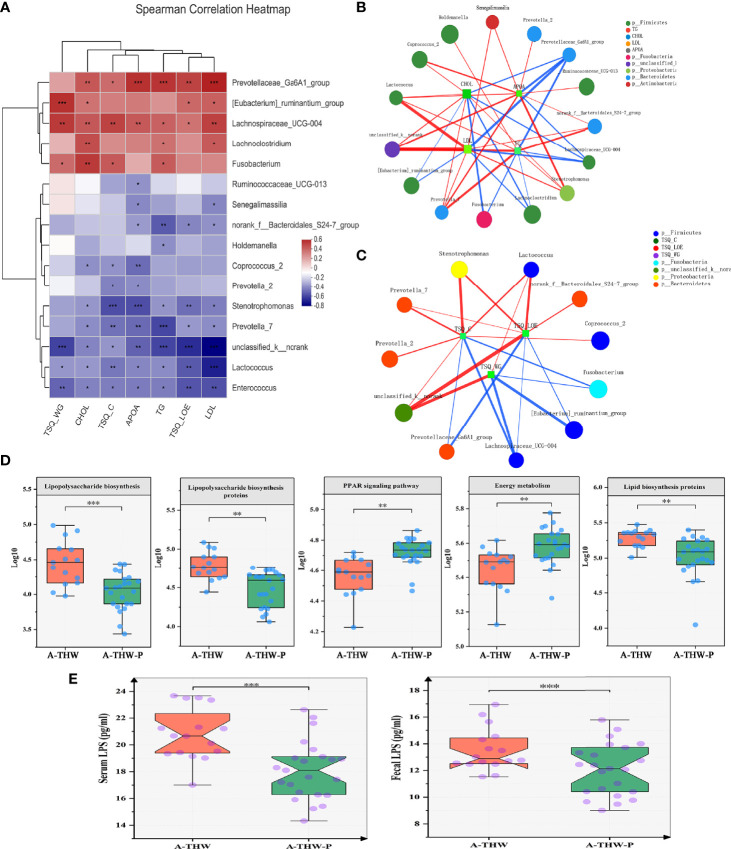
Spearman correlation analysis of environmental factors and characteristics of gut microbiota function. **(A)** The relationships among seven clinical indicators and 16 differentially abundant genera (**Figure 4C**) were estimated using Spearman correlation analysis, and the results **(B, C)** are shown in co-correlation networks. Color intensity represents the magnitude of correlations. Red, positive correlations; blue, negative correlations. Spearman correlation coefficient values below −0.3 (negative correlation) are indicated as red edges, and coefficient values above 0.3 (positive correlation) are indicated as green edges. The node size shows the gene number for each species. The five typically differentially abundant KEGG pathways **(D)** and plasma and fecal LPS levels **(E)** for the A-THW and A-THW-P groups. Wilcoxon rank-sum test. A-THW, after treatment with THW plus a placebo; A-THW-P, after treatment with THW plus the probiotic. *P-value < 0.05; **P-value < 0.01; ***P-value < 0.001.

A correlation heatmap was generated to further demonstrate the relationship between the relative abundance of different genera (n = 16) and clinical features (n = 7) (P < 0.05, |correlation coefficient| > 0.3, **Figure 5A**). Correlation networks were generated further to demonstrate the above results (**Figures 5B, C**). Bacteria enriched in the A-THW-P group were negatively correlated with plasma lipid values (CHOL, TG, LDL, and Apo A). The bacteria enriched in the A-THW group were positively correlated with these values. The abundances of the A-THW group-enriched genera, including *Fusobacterium*, *[Eubacterium]_ruminantium_group*, and *Parasutterella*, were positively correlated with TSQ-WG and TSQ-LOE values. The abundances of the A-THW-P group-enriched genera, including *Coprococcus_2*, were negatively correlated with TSQ-C values. However, TSQ-C values were positively correlated with some of the A-THW group-enriched genera, including *Fusobacterium*. This finding suggests that the gut microbiota may be involved in the occurrence of the above complications and dyslipidemia.

### Functional Alterations in the Gut Microbiota With Probiotics

We carried out PICRUSt analysis to annotate the functions of the microbiota and explore how the microbiota caused complications and dyslipidemia. KEGG Pathways (level 3) of energy metabolism and PPAR signaling pathway were significantly enriched in the A-THW-P group compared with those in the A-THW group (P < 0.05, **Figure 5D**). Pathways of lipopolysaccharide (LPS) biosynthesis, LPS biosynthesis proteins, and lipid biosynthesis proteins were enriched in the A-THW group (P < 0.05, **Figure 5D**). We observed that fecal/plasma LPS levels of patients in the A-THW group increased simultaneously than those of the A-THW-P group (P < 0.001, **Figure 5E**), which is consistent with the enhancement of metabolic pathways related to LPS synthesis (**Figure 5D**). As mentioned above, we identified the changes in the gut microbiota composition, and the abundance of different species was correlated with some plasma lipid levels (e.g., CHOL, TG, LDL, and Apo A) and symptom scores (e.g., TSQ-LOE, TSQ-WG, and TSQ-C). Moreover, we found that the probiotics also improved the function of the gut microbiota. Therefore, we hypothesized that probiotic administration can improve complications, that this effect is related to the recovery of the gut microbiota, and that microbiota metabolism might be a regulatory factor.

### The Effect of Probiotics on the Oral Microbiota of Patients With THW

**Figure 6:**
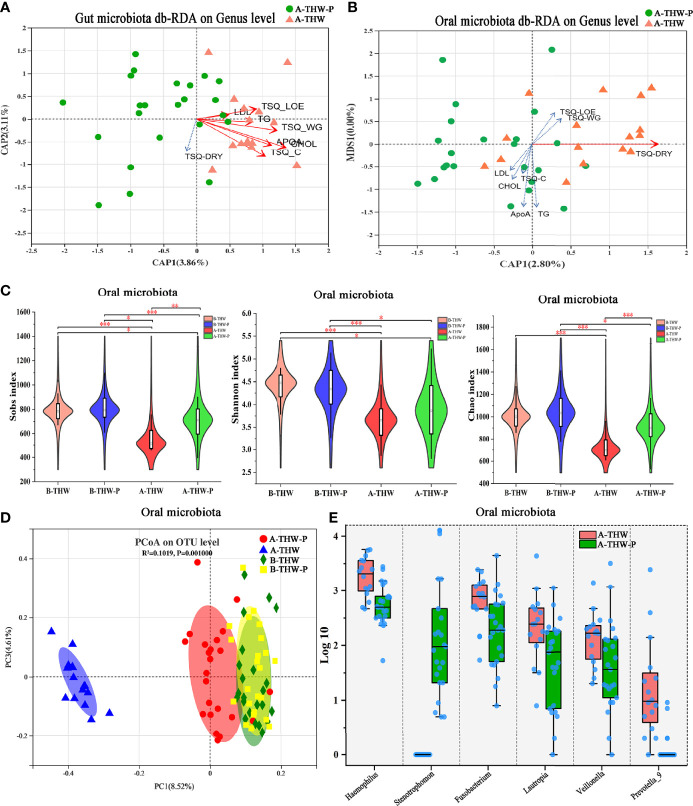
The shift in the oral microbiota composition in patients with or without probiotic administration. dB-RDA shows the relationship of environmental factors to the gut **(A)** and oral **(B)** microbial community structures. **(C)** The Sobs, Chao, and Shannon indexes of the B-THW, B-THW-P, A-THW, and A-THW-P groups were compared. **(D)** Binary-chord principal component analysis; the oral microbiotas of people from the B-THW, B-THW-P, A-THW, and A-THW-P groups were significantly different. **(E)** Comparisons of the oral microbiota relative abundance at the genus level in the A-THW and A-THW-P groups by Mann–Whitney U-tests (P < 0.05). B-THW, before the treatment of THW plus a placebo; B-THW-P, before the treatment of THW plus the probiotic combination; A-THW, after treatment with THW plus a placebo; A-THW-P, after treatment with THW plus the probiotic. *P-value < 0.05; **P-value < 0.01; ***P-value < 0.001.

**Figure 7:**
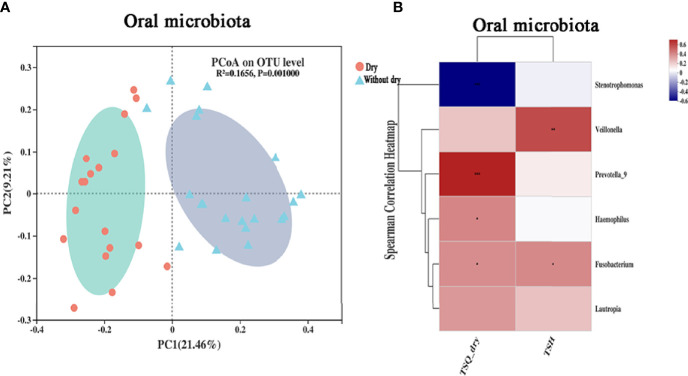
Correlation analysis of the oral microbiota and TSQ-DRY scores. **(A)** Unweighted UniFrac principal component analysis; the oral microbiotas of people from the dry and without dry groups were significantly different. **(B)** The relationships among 2 clinical indicators and 6 differentially abundant genera (**Figure 6E**) were estimated using Spearman correlation analysis. * P-value <0.05; ** P-value <0.01; *** P-value <0.001.

The results of distance-based redundancy analysis (dB-RDA) (**Figure 6A**) showed that there is no correlation between TSQ-DRY and gut microbiota, while TSQ-DRY had a significant influence on the oral microbiota (**Figure 6B**). Therefore, we observed improved oral microbiota due to the probiotics and explored its relationship with TSQ-DRY. The alpha diversities were measured by the observed Sobs, Chao, and Shannon indexes. Compared to those in the A-THW group, the Sobs and Chao index values were significantly higher in the A-THW-P group (All, P < 0.05, **Figure 6C**). The Shannon index showed a rising trend with an increasing trend in Probiotics supplementation. Still, there was no significant difference (P > 0.05, **Figure 6C**). The alpha diversities (Sobs, Chao, and Shannon) of patients from the A-THW-P group returned to the levels of the B-THW/B-THW-P groups compared with the A-THW group (**Figure 6C**). The same trend was observed in beta diversity measured by binary-chord analysis (**Figure 6D**). Similar to the observation for the gut microbiota, the probiotics significantly reduced the MDI value of the oral microbiota of patients in the A-THW-P group compared to those of the A-THW group (**Supplementary Figure 2D**). The microbial composition of each group is presented at the genus level (**Supplementary Figure 2C**). The dominant genera of the oral microbiota in the A-THW-P group included *Stenotrophomonas* and *Veillonella* (**Figure 6E**). *Haemophilus*, *Fusobacterium, Lautropia, and Prevotella_9* were enriched in the A-THW group (**Figure 6E**). In addition, we regrouped the A-THW-P/A-THW group according to the occurrence of dry mouth, and beta diversity analysis based on the unweighted unifrac distance showed that the samples of the dry mouth group significantly deviated from those of the group without a dry mouth (**Figure 7A**, P < 0.05). For investigation of correlations between the microbiota and TSQ-DRY, correlation analysis showed that the levels of *Prevotella_9, Haemophilus, Fusobacterium*, and *Lautropia* in the oral cavity were positively correlated with TSQ-DRY scores. In contrast, *Stenotrophomonas* abundance was negatively correlated with TSQ-DRY scores (**Figure 7B**).

## Discussion

The purpose of this study was to assess the impact of probiotics on complications and dyslipidemia in DTC patients with THW. First, we found that probiotics could reduce the incidence of lack of energy, weight gain, constipation, and dry mouth and reduce plasma lipid levels. However, they did not significantly improve the incidence of edema. A significant reduction in fecal/plasma LPS levels and altered oral and gut microbiota were observed, which may account for the protective effect of probiotics supplementation.

Previous studies have shown that variation in thyroid function can affect the gut microbiota (22). In this study, DTC patients with THW had lower gut microbiota diversity (alpha diversity) than before THW treatment. This finding is consistent with a previous study investigating the gut microbiota in Hashimoto’s thyroiditis patients with hypothyroidism (23). We found that probiotics could improve the diversity of the gut microbiota.

THW can cause transient dyslipidemia, which will increase the incidence of cardiovascular disease and pancreatitis in DTC patients during THW (24, 25). The range of changes in blood lipids caused by THW is vast: in some patients, the level of change is very slight, while in others, severe hypercholesterolemia is observed. This situation has also been observed in patients with primary thyroid dysfunction (26). The occurrence of dyslipidemia in the THW period cannot be explained by a single mechanism. Several studies have found that TSH and thyroid hormone levels are independently related to total cholesterol levels; in addition, various clinical factors, including gender, age, fasting blood glucose, and BMI, are also considered to have independent effects on total cholesterol levels (27). There is currently no article to evaluate the influence of gut microbiota on blood lipid levels during THW. In our study, plasma lipid levels (e.g., CHOL, TG, HDL, and ApoA) were positively correlated with gut microbiota constituent abundances (e.g., *Coprococcus_2*, and *norank_f:Bacteroidales_S24-7_group*, etc.). According to previous reports, the above microbiota constituents can participate in the production of short-chain fatty acids (SCFAs) (28). One study has shown that SCFAs could regulate plasma lipid metabolism (29). In addition, the lipid biosynthesis protein pathway of the gut microbiota was significantly reduced, and the PPAR metabolism pathway was upregulated after the application of probiotics, according to PICRUSt analysis. According to the report, the PPAR pathway can participate in mediating lipid metabolism (30). We speculated that probiotics might improve the plasma lipid level of patients with THW through the PPAR metabolism and lipid biosynthesis pathway. The accumulation of plasma CHOL, LDL, and other lipids can lead to obesity (31). This effect may be related to the improvement produced by probiotics in weight gain complications.

In our study, we found that probiotic administration significantly reduced patients’ fecal LPS and plasma LPS concentrations. The abundance of LPS-producing bacteria (e.g., *Fusobacterium*) and LPS synthesis-related pathways were also considerably reduced, which may explain the reduction in fecal LPS concentrations by probiotics. There is a correlation between the fecal/plasma LPS level and the impaired intestinal barrier (32). When intestinal permeability increased, LPS in fecal could pass through the intestine and enter the circulation. Probiotics such as *Lactobacillus* and *Bifidobacterium* protect the gut barrier by increasing tight junction protein expression (e.g., occludin and claudin 3) and reducing inflammatory markers (32–34). Therefore, probiotics may reduce serum LPS level by regulating the intestinal barrier in DTC patients with THW. LPS can bind to Toll-like receptors on thyroid cells and affect the expression of thyroglobulin and sodium iodine transporter (35, 36). This activity may further affect sensitivity to subsequent RAI therapy.

We found that probiotics can reduce the incidence of lack of energy. Patients mainly showed fatigue complications, which may be related to improving the gut microbiota produced by probiotics. A study analyzed chronic fatigue syndrome patients’ fecal and plasma samples and healthy volunteers (15). The results showed that compared with healthy people, the bacterial diversity of patients with chronic fatigue syndrome was significantly reduced, and the number of types of anti-inflammatory bacteria was considerably reduced. In addition, bacteria in the intestine can enter the plasma through the damaged intestinal barrier, worsening the disease. The application of probiotics, especially *Bifidobacterium infantis 35624*, can improve the gut microbiota and mucosal barrier function and reduce proinflammatory cytokines and inflammatory biomarkers to delay the progression of chronic fatigue syndrome (37, 38). In our study, probiotic administration increased the alpha diversity of the gut microbiota and reduced the abundance of inflammatory bacteria, such as *Fusobacterium*, while enriching the energy metabolism pathway. Additionally, probiotic administration reduced the fecal and plasma LPS levels, suggesting that probiotics may improve intestinal barrier function. This evidence shows that probiotics reduce the incidence of fatigue in patients with THW, which may be related to the improvement in microbiota diversity, intestinal inflammation, barrier function, and reduction in inflammatory bacterial abundance. In addition, this study showed that probiotic administration could significantly reduce the incidence of constipation in patients with THW. Correlation analysis showed that the occurrence of constipation was negatively correlated with *Prevotella_2*, *Prevotella_7*, and *Lactococcus* abundance, suggesting that probiotics may improve constipation by increasing the abundance of the above bacteria, which is consistent with previous studies (39).

This study showed that probiotics could increase the alpha diversity index of the oral microbiota of patients with THW and reduce the abundances of *Hemophilus, Fusobacterium*, *Lautropia*, and *Prevotella_9*. Compared with healthy volunteers, those with hypothyroidism during pregnancy had significantly higher *Prevotella* abundance (40). In our study, probiotic administration reduced the incidence of dry mouth complications. Beta diversity analysis showed that oral samples from patients with dry mouth complications significantly deviate from those without dry mouth complications. Correlation analysis showed that *Prevotella_9*, *Haemophilus*, and *Fusobacterium* were positively correlated with the occurrence of dry mouth complications. It is reported that LPS produced by oral bacteria such as *Fusobacterium* may cause a decrease in mucin synthesis in salivary acinar cells, which is accompanied by acinar cell apoptosis (41), this may be a potential mechanism for oral bacteria to affect the occurrence of dry mouth. In addition, these oral bacteria were significantly reduced by probiotic administration. We speculate that this effect may be related to the improvement in the microbiota yielded by probiotic administration. Unfortunately, this study did not measure the salivary gland flow rate of patients with dry mouth complications to reflect their salivary gland function. In the future, additional studies are needed to clarify the relationship between oral bacteria and the occurrence of dry mouth complications in patients with THW.

Iodothyronine-deiodinases play a vital role in the conversion of thyroxine (T4) to its active form triiodothyronine (T3) or reverse T3, its inactive form (42). Deiodinase activity has been found in the gut (43, 44); the presence of gut microbiota might be binding to T3, reducing or eliminating deiodinase activity (43, 44). One study showed that gavage of probiotic yogurt significantly increased serum T3 levels in rats (45). The presence of beneficial bacteria such as probiotics in the gut may accelerate the conversion of T4 to T3 (45–47). In addition, β-glucuronidases and sulfatases enzymes can hydrolyze glucuronide and iodothyronine sulfate metabolites, thereby inactivating thyroid hormones in the liver (35). Gut microbiota expresses β-glucuronidases and sulfatases enzymes (35). Probiotics may affect the activity of these enzymes by modulating the gut microbiota. Although not statistically significant, fT3 levels were higher in the patients treated with probiotics in our study, and this result may be due to probiotics affecting the deiodination of thyroid hormone or the activity of β-glucuronidases and sulfatases enzymes. Many researchers believe that the complications during THW are related to hypothyroidism caused by thyroid hormone deficiency. Since probiotics did not significantly change thyroid hormone levels but did improve complications, we infer that thyroid hormone deficiency during THW may shape a dysbiosis microbiota and cause increased complications. Probiotics can reduce complications by improving dysbiosis microbiota. In addition, probiotics could also be able to prevent serum hormonal fluctuations (48). It is worth noting that iodine levels have been proven to influence the gut microbiota (42). A low-iodine diet and changes in thyroid hormone levels during THW may jointly participate in microbiota transformation.

In addition, although to our knowledge, this study is the first randomized controlled trial showing that DTC patients may have a dysbiosis gut and oral microbiota during THW, and probiotics administration may reduce complications and dyslipidemia in patients after THW by improving the oral and gut microbiota, it must be noted that there were several limitations of this study. To ensure the scientificity and reliability of this study, we implemented strict inclusion and exclusion criteria, which can lead to a dramatic reduction in the number of patients enrolled in the study. This study’s sample size is limited. And the subjective evaluation scales and standardized collection of data may lead to potential observer bias. Nevertheless, in most current research on THW-related complications, the evaluation scales are the mainstream evaluation method, and its assessment of complications is relatively reliable (11, 49). There are hardly any other objective evaluation methods in this field. In the future, more effective evaluation methods need to be developed, such as a combination of evaluation scales and laboratory tests. Compared to 16S rDNA amplicon sequencing, shotgun generation sequencing metagenomics has more power to identify a larger number of species. The results of this study are limited, and no major clinical consequences can be proven at this stage. The possible role of probiotics as “adjuvants for THW treatment” may become a starting point for probiotic researchers and endocrinologists to clarify the interaction between the endocrine system and intestinal and oral microecology. So far, this correlation has not been evaluated. Researchers should explore other probiotic strains or longer follow-up times and larger sample sizes in the future.

## Data Availability Statement

The data presented in the study are deposited in the NCBI BioProject repository, https://www.ncbi.nlm.nih.gov/, accession number PRJNA784752.

## Ethics Statement

The Ethics Committee approved all protocols applied in this study at the First Affiliated Hospital of Harbin Medical University (Eth. 201816). The patients/participants provided their written informed consent to participate in this study.

## Author Contributions

Guarantee all integrity associated with this study, YW, BL, and FZ. Study concepts/study design or data acquisition or data analysis/interpretation, BL and FZ. Manuscript drafting or manuscript revision for important intellectual content, all authors. Agree to ensure any questions related to the work are appropriately resolved, all authors. Agree to be accountable for the content of the work, all authors. Collect the samples, YLiu, YLu, XW, JF, XJ, WY, and XG. Perform the bioinformatics and statistical analyses and interprets the data, SS, ZL, LL, HC, HW, and SW. Revise the manuscript for important content, YW. All authors contributed to the article and approved the submitted version.

## Funding

This research was funded by the National Natural Science Foundation of China grants (NSFC81970466).

## Conflict of Interest

The authors declare that the research was conducted in the absence of any commercial or financial relationships that could be construed as a potential conflict of interest.

## Publisher’s Note

All claims expressed in this article are solely those of the authors and do not necessarily represent those of their affiliated organizations, or those of the publisher, the editors and the reviewers. Any product that may be evaluated in this article, or claim that may be made by its manufacturer, is not guaranteed or endorsed by the publisher.
