# Supplementary material for: Randomized Clinical Trial: Probiotics Alleviated Oral-Gut Microbiota Dysbiosis and Thyroid Hormone Withdrawal-Related Complications in Thyroid Cancer Patients Before Radioiodine Therapy Following Thyroidectomy
Source: Front Endocrinol (Lausanne). 2022 Mar 8;13:834674. doi: 10.3389/fendo.2022.834674 (PMC8958007; doi:10.3389/fendo.2022.834674)
Supplement: Supplementary file 1 [file DataSheet_1.docx]

Supplementary Material

# Supplementary Data

## Exclusion criteria

Received prior radioiodine therapy or had known metastatic disease; positive thyroid peroxidase antibodies (TPOAb) or thyroglobulin antibodies (TGAb); underlying thyroid dysfunction; ongoing therapies interfering with levothyroxine absorption and/or metabolism (i.e., sucralfate, aluminum-containing antacids, and bile acids sequestrants); pregnancy; lactation; cigarette smoking; alcohol addiction; severe periodontal disease; cardiovascular and metabolic diseases, such as hypertension, diabetes mellitus, and BMI>27; recent (<3 months prior) use of lipid-lowering agents, antibiotics, hormonal medication, laxatives, proton pump inhibitors, insulin sensitizers or traditional Chinese medicine; known history of disease with an autoimmune component, such as multiple sclerosis, rheumatoid arthritis, irritable bowel syndrome, or IBD; and history of malignancy or any gastrointestinal tract surgery (e.g., gastrointestinal surgery, cholecystectomy or appendectomy).

## Plasma and fecal sample collection

All participants were examined in the morning after an overnight fast (≥8 h). Peripheral plasma (15 mL) was collected from all subjects and stored in corresponding plasma collection tubes at 4 °C for the detection of thyroid function indicators (free triiodothyronine, [fT3], free thyroxine, [fT4], and thyrotropin, [TSH], thyroglobulin, [Tg]) examinations, liver function (alanine aminotransferase [ALT], aspartate transaminase [AST], album [ALB], total protein [TP], globulin [GLB), total bilirubin [TBIL], direct bilirubin [DBIL], indirect bilirubin [IBIL], gamma-glutamyl transpeptidase [GGT], alkaline phosphatase [AKP], lactate dehydrogenase [LDH], blood urea nitrogen [BUN], creatinine [Cr], uric acid [UA], glucose [GLU]), plasma lipid (total cholesterol [CHOL], triacylglycerol [TG], low-density lipoprotein [LDL], apolipoprotein A [ApoA], high-density lipoprotein [HDL], very low density lipoprotein [VLDL], apolipoprotein B [ApoB], lipoprotein(a) [Lpa]) and serum LPS analyses. Participants were prohibited from eating, drinking other liquids than water, and doing oral hygiene activities at least 1 h before sampling. From each patient, oral washings were collected for subsequent 16S rRNA gene analysis. Concurrently, all subjects were provided Commode Specimen Collection Kits for feces collection, and samples were sent to our laboratory on an ice pack within two hours. Each fecal sample was immediately divided into aliquots, frozen on dry ice, and stored at -80 °C for subsequent 16S rRNA gene and LPS analysis.

## Analysis of clinical parameters

Serum fT3, fT4, TSH, and Tg levels were measured by a chemiluminescent immunoassay (Abbott Diagnostics, Tokyo, Japan). Liver function and plasma lipid indexes were assessed using an automated biochemistry analyzer (Beckman Coulter, California, USA) and auxiliary reagents. Serum and fecal supernatant LPS indicators were measured with corresponding ELISA kits (Shanghai Jiang Lai Biotechnology Co., Ltd., Shanghai, China), following the manufacturer's instructions.

# Supplementary Figures and Tables

## Supplementary Figures


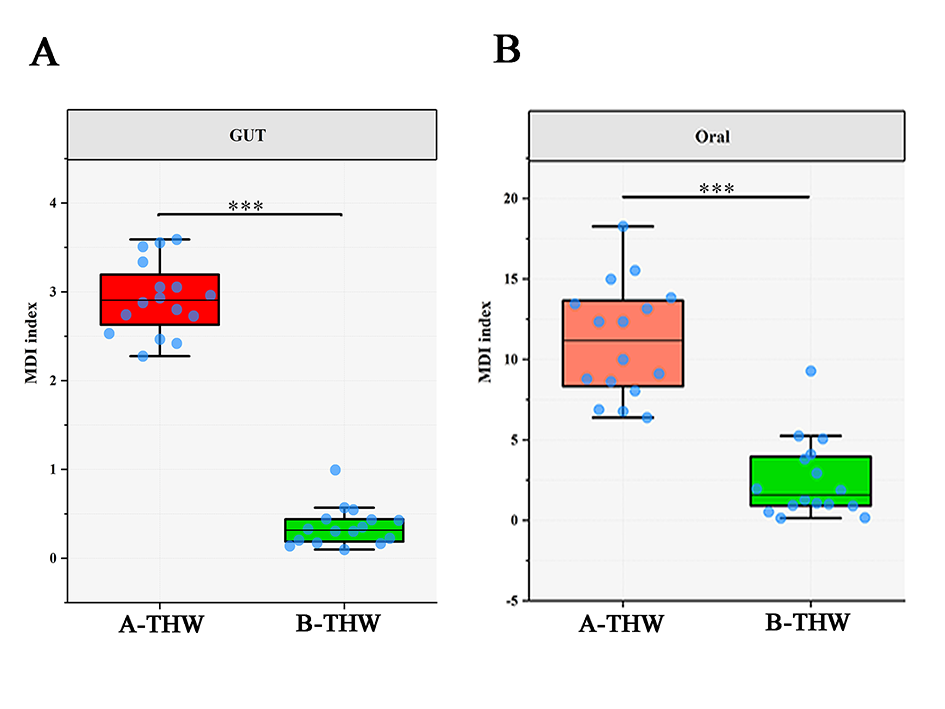


**Supplementary Figure 1.** Transitions in MDI index of gut (A) and oral (B) microbiome of DTC patients following THW. B-THW, before the treatment of THW plus a placebo; A-THW, after treatment with THW plus a placebo. * P-value <0.05; ** P-value <0.01; *** P-value <0.001.

**
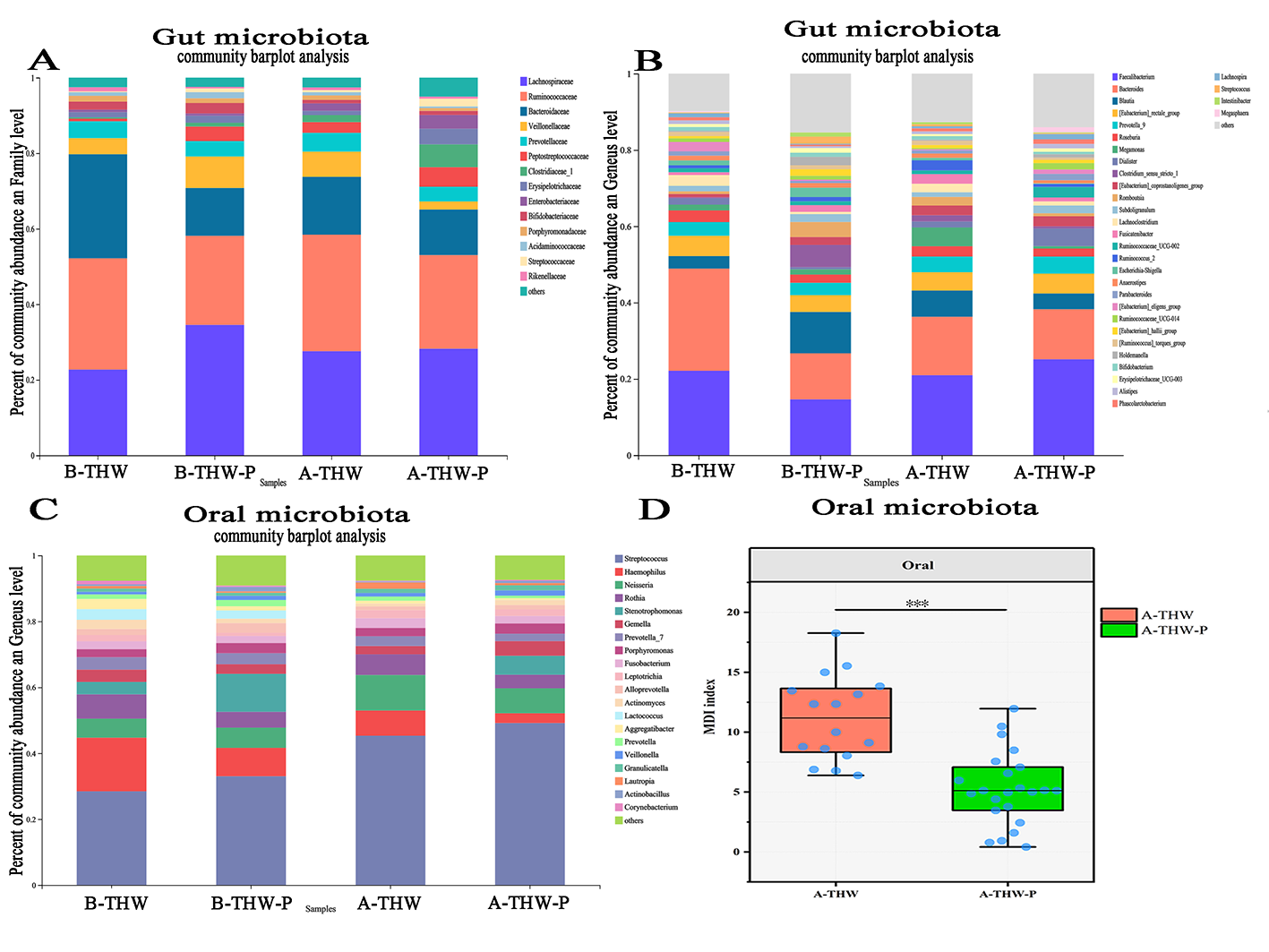
**

**Supplementary Figure 2. Oral and gut microbiota composition and MDI.** The relative abundances of the gut bacterial family (A) and genera (B) and the oral bacterial genera (C) clustered into different groups were determined, revealing that the microbiota compositions differed significantly. (D) Box plot showing the MDI of the oral microbiota in the A-THW and A-THW-P groups. B-THW, before the treatment of THW plus a placebo; B-THW-P, before the treatment of THW plus the probiotic combination; A-THW, after treatment with THW plus a placebo; A-THW-P, after treatment with THW plus the probiotic. MDI, microbial dysbiosis index; * P-value <0.05; ** P-value <0.01; *** P-value <0.001.

# Supplementary Table

**Supplementary Table 1. Plasma indicators.**

| **Table-S1. Plasma indicators** | | | |
| --- | --- | --- | --- |
| **Variable** | **Placebo group (n=16)** | **Probiotic group (n=23)** | **P value** |
| **Hepatic function** | | | |
| ALT (U/L, mean±SD) | | | |
| Baseline | 16.51±8.77 | 14.36±5.72 | 0.741 |
| End of intervention (week 4) | 28.38±16.57 | 26.57±12.87 | 0.099 |
| AST (U/L, mean±SD) | | | |
| Baseline | 19.73±4.63 | 17.67±3.33 | 0.187 |
| End of intervention (week 4) | 30.28±13.71 | 24.48±12.74 | 0.361 |
| ALB (g/L, mean±SD) | | | |
| Baseline | 44.67±2.15 | 45.33±2.86 | 0.471 |
| End of intervention (week 4) | 44.25±3.06 | 44.56±4.02 | 0.703 |
| TP (g/L, mean±SD) | | | |
| Baseline | 73.71±3.41 | 74.10±3.78 | 0.662 |
| End of intervention (week 4) | 75.39±4.82 | 75.78±7.00 | 0.322 |
| GLB (g/L, mean±SD) | | | |
| Baseline | 28.93±3.31 | 28.72±3.51 | 0.801 |
| End of intervention (week 4) | 30.45±3.82 | 29.36±3.58 | 0.329 |
| TBIL (µmol/L, mean±SD) | | | |
| Baseline | 11.69±2.66 | 12.84±3.72 | 0.217 |
| End of intervention (week 4) | 11.41±3.64 | 11.66±3.30 | 0.927 |
| DBIL (µmol/L, mean±SD) | | | |
| Baseline | 2.17±0.57 | 2.26±0.63 | 0.724 |
| End of intervention (week 4) | 2.33±1.58 | 2.35±1.54 | 0.713 |
| IBIL (µmol/L, mean±SD) | | | |
| Baseline | 2.17±0.57 | 2.26±0.63 | 0.724 |
| End of intervention (week 4) | 9.04±2.56 | 9.31±2.23 | 0.843 |
| GGT (µmol/L, mean±SD) | | | |
| Baseline | 23.14±15.07 | 23.15±13.59 | 0.703 |
| End of intervention (week 4) | 21.98±9.18 | 25.24±7.02 | 0.307 |
| AKP (µmol/L, mean±SD) | | | |
| Baseline | 68.27±20.04 | 62.93±16.93 | 0.315 |
| End of intervention (week 4) | 71.57±18.46 | 79.26±20.89 | 0.446 |
| LDH (µmol/L, mean±SD) | | | |
| Baseline | 156.22±21.08 | 147.93±25.90 | 0.196 |
| End of intervention (week 4) | 168.59±46.32 | 171.01±42.02 | 0.286 |
| BUN (µmol/L, mean±SD) | | | |
| Baseline | 4.57±0.75 | 4.34±1.22 | 0.196 |
| End of intervention (week 4) | 4.42±1.49 | 4.39±1.23 | 0.670 |
| CR (µmol/L, mean±SD) | | | |
| Baseline | 60.94±12.79 | 57.95±10.48 | 0.544 |
| End of intervention (week 4) | 62.00±10.96 | 66.00±14.86 | 0.247 |
| UA (µmol/L, mean±SD) | | | |
| Baseline | 276.04±55.39 | 268.10±50.70 | 0.641 |
| End of intervention (week 4) | 290.67±63.37 | 296.01±53.86 | 0.522 |
| GLU (µmol/L, mean±SD) | | | |
| Baseline | 5.00±0.37 | 5.21±0.31 | 0.057 |
| End of intervention (week 4) | 5.25±0.39 | 5.19±0.97 | 0.185 |

**Supplementary Table 1. Plasma indicators.** The measurement data and enumeration data were statistically analyzed with t-test (or one-way ANOVA for multi-group comparison) and χ2 test, respectively. Measurement data are expressed as the mean ± SD. ALT, alanine aminotransferase; AST, aspartate transaminase; ALB, album; TP, total protein; GLB, globulin; TBIL, total bilirubin; DBIL, direct bilirubin; IBIL, indirect bilirubin; GGT, gamma-glutamyl transpeptidase; AKP, alkaline phosphatase; LDH, lactate dehydrogenase; BUN, blood urea nitrogen; Cr, creatinine; UA, uric acid; GLU, glucose; and SD, standard deviation. P-value < 0.05 was considered significant. * P-value <0.05

| **Supplementary Table 2. Pyrosequencing characteristic and α Diversity.** | | | | | | |
| --- | --- | --- | --- | --- | --- | --- |
| **Sample/** | **Sobs** | **Ace** | **Chao** | **Shannon** | **Coverage** | **Invsimpson** |
| **Estimators** | **Mean** | **Mean** | **Mean** | **Mean** | **(%)** | **Mean** |
| B-THW group (oral) | 519 | 636.720 | 637.207 | 4.0191 | 0.9974 | 24.8803 |
| B-THW-P group (oral) | 468 | 584.867 | 584.492 | 3.9205 | 0.9976 | 21.1311 |
| A-THW group (oral) | 557 | 769.34 | 736.96 | 3.6351 | 0.9953 | 15.808 |
| A-THW-P group (oral) | 708 | 933.5 | 930.37 | 3.9181 | 0.99407 | 21.728 |
| B-THW group (gut) | 468 | 584.87 | 584.49 | 3.92045 | 0.99757 | 21.131 |
| B-THW-P group (gut) | 519 | 636.722 | 637.2 | 4.019 | 0.997354 | 24.88 |
| A-THW group (gut) | 406 | 551.01 | 524.11 | 3.5437 | 0.99758 | 16.808 |
| A-THW-P group (gut) | 496.48 | 629.88 | 625.57 | 3.8994 | 0.99733 | 22.12 |

**Supplementary Table 2. Pyrosequencing characteristic and α Diversity.** B-THW, before the treatment of THW plus a placebo; B-THW-P, before the treatment of THW plus the probiotic combination; A-THW, after treatment with THW plus a placebo; A-THW-P, after treatment with THW plus the probiotic.
